# Diagnostic value of dual-layer spectral detector CT parameters for differentiating high- from low-grade bladder cancer

**DOI:** 10.1186/s13244-024-01881-8

**Published:** 2025-01-02

**Authors:** Li Chen, Lili Xu, Xiaoxiao Zhang, Jiahui Zhang, Xin Bai, Qianyu Peng, Erjia Guo, Xiaomei Lu, Shenghui Yu, Zhengyu Jin, Gumuyang Zhang, Yi Xie, Huadan Xue, Hao Sun

**Affiliations:** 1 Department of Radiology, State Key Laboratory of Complex Severe and Rare Disease, Peking Union Medical College Hospital, Chinese Academy of Medical Sciences and Peking Union Medical College, Beijing, People’s Republic of China; 2 Department of Radiology, Zhejiang Cancer Hospital, Hangzhou Institute of Medicine (HIM), Chinese Academy of Sciences, Hangzhou, People’s Republic of China; 3 CT Clinical Science, Philips Healthcare, Shenyang, People’s Republic of China; 4 CT Clinical Science, Philips Healthcare, Beijing, People’s Republic of China; 5 National Center for Quality Control of Radiology, Beijing, People’s Republic of China; 6 Department of Urology, State Key Laboratory of Complex Severe and Rare Disease, Peking Union Medical College Hospital, Chinese Academy of Medical Sciences and Peking Union Medical College, Beijing, People’s Republic of China

**Keywords:** Urinary bladder neoplasms, Pathology, Spectral CT, Computed tomography urography, Arterial enhancement fraction

## Abstract

**Objectives:**

This study aimed to investigate the diagnostic value of spectral parameters of dual-layer spectral detector computed tomography (DLCT) in distinguishing between low- and high-grade bladder cancer (BCa).

**Methods:**

This single-center retrospective study included pathologically confirmed BCa patients who underwent preoperative contrast-enhanced DLCT. Patients were divided into low- and high-grade groups based on pathology. We measured and calculated the following spectral CT parameters: iodine density (ID), normalized ID (NID), arterial enhancement fraction (AEF), extracellular volume (ECV) fraction, virtual non-contrast (VNC), slope of the attenuation curve, and Z effective (Z_eff_). Univariate and multivariable logistic regression analyses were used to determine the best predictive factors in differentiating between low- and high-grade BCa. We used receiver operating characteristic curve analysis to assess diagnostic performance and decision curve analysis to determine the net benefit.

**Results:**

The study included 64 patients (mean age, 64 ± 11.0 years; 46 men), of whom 42 had high-grade BCa and 22 had low-grade BCa. Univariate analysis revealed that differences in ID and NID in the corticomedullary phase, AEF, ECV, VNC, and Z_eff_ images were statistically significant (*p* = 0.001–0.048). Multivariable analysis found that AEF was the best predictor of high-grade tumors (*p* = 0.006). With AEF higher in high-grade BCa, AEF results were as follows: area under the curve (AUC), 0.924 (95% confidence interval, 0.861–0.988); sensitivity, 95.5%; specificity, 81.0%; and accuracy, 85.9%. The cutoff valve of AEF for predicting high-grade BCa was 67.7%.

**Conclusion:**

Using DLCT AEF could help distinguish high-grade from low-grade BCa.

**Critical relevance statement:**

This research demonstrates that the arterial enhancement fraction (AEF), a parameter derived from dual-layer spectral detector CT (DLCT), effectively distinguishes between high- and low-grade bladder cancer, thereby aiding in the selection of appropriate clinical treatment strategies.

**Key Points:**

- This study investigated the value of dual-layer spectral detector CT in the assessment of bladder cancer (BCa) histological grade.
- The spectral parameter arterial enhancement fraction could help determine BCa grade.
- Our results can help clinicians formulate initial treatment strategies and improve prognostications.

**Graphical Abstract:**

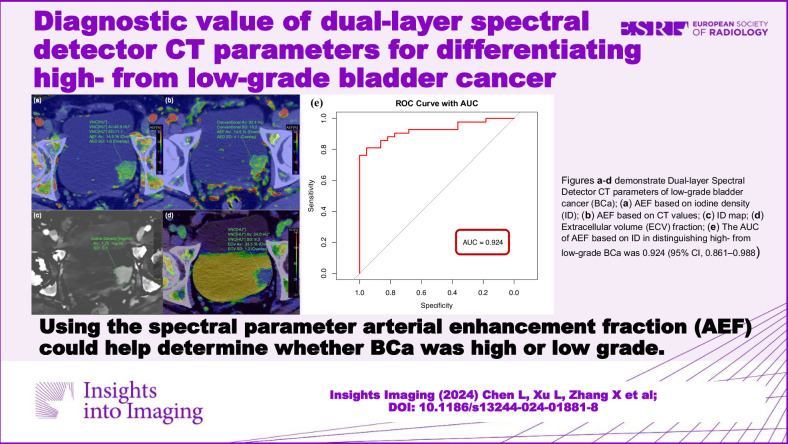

## Introduction

Bladder cancer (BCa) is the most common type of malignancy in the urinary tract and ranks among the most prevalent cancers globally [1, 2]. Based on histological differences, BCa is categorized as a low- or high-grade tumor. Distinguishing between low- and high-grade BCa is critical in diagnosis and treatment decision-making for each patient [3–5]. High-grade BCa has a higher recurrence rate and is more likely to progress to muscle invasion. Low-grade BCa patients are recommended to undergo one immediate intravesical chemotherapy after transurethral resection of bladder tumor [6]. For patients with high-grade tumors, additional instillation of Bacillus Calmette–Guérin might be required for 1–3 years, and radical cystectomy might also be necessary [4].

Methods currently used to detect BCa include imaging techniques such as ultrasound, computed tomography (CT), magnetic resonance imaging, and positron emission tomography/CT; as well as cystoscopy, biopsy, and cytology [7–9]. BCa management depends heavily on pathological grade, but analyses of cystoscopic biopsies sometimes underestimate the grade [10]. Recent authoritative guidelines and compelling review articles highlight the indispensable role of CT as a cornerstone in the diagnosis and management of BCa. European Association of Urology guidelines recommend that CT urography (CTU) be used during initial workup for patients with hematuria and suggest its use once a bladder tumor has been detected in selected cases (e.g., tumors located in the trigone; multiple or high-risk tumors) [4]. American Urological Association guidelines recommend CT as the imaging modality during workup for BCa [11]. A review published in *The Lancet* affirmed CTU’s role in the diagnosis of BCa; the sensitivity (Sen) of CTU in detecting BCa is 0.87, while its specificity (Spc) is 0.99 [12]. Another review published in the *British Medical Journal* mentions CTU as the first choice for suspected BCa [13]. It is particularly useful for visualizing the entire urinary tract and identifying potential sites of malignancy [14].

Using conventional CT, clinicians can evaluate bladder tumors by morphological features, density, and enhancement features. A previous study has confirmed that CT features can be used to assess risk stratification of non–muscle-invasive BCa [15]. Therefore, evaluating CT features to predict the pathological grade of BCa before surgery is feasible, yet determining its histological grade remains challenging without definitive quantitative metrics [16, 17].

Dual-energy CT (DECT) is an advanced technology that enables the acquisition of both conventional and dual-energy sequences. Using DECT, clinicians can characterize tissue based on the unique attenuation properties of each substance within the tissue at different energies, as well as based on conventional Hounsfield unit (HU) values [18]. Dual-layer spectral detector CT (DLCT) uses a dual-layer detector to convert high- and low-energy X-rays simultaneously and a stereo system to acquire the data. This enables simultaneous, isotropic, homogeneous, and synchronized separation of energy signals, resulting in a broader range of virtual single-energy images (MonoE) and additional parameters, such as an effective atomic number (Z_eff_) [19, 20]. Many studies have demonstrated the potential of DLCT parameters to help in tumor diagnosis. Specifically, Nagayama et al found that non-enhanced DLCT parameters help distinguish adrenal adenomas from non-adenomas [21]. Additionally, Zhou and colleagues discovered that the DLCT parameter extracellular volume (ECV) holds great value in predicting lymph node metastasis in papillary thyroid carcinoma [22]. Zhu et al found that ECV can help predict microsatellite instability status and prognosis in locally advanced gastric cancer [23]. Wang et al found that using iodine density (ID) and Z_eff_ can help differentiate the pathologies of pancreatic neuroendocrine neoplasms [24]. Chen et al used DLCT metrics to predict the pathological stage and histological grade of colorectal adenocarcinoma [25]. Nevertheless, no study has yet been conducted on pathological grading of BCa based on DLCT parameters.

Therefore, in this study, we aimed to investigate the diagnostic value of spectral parameters of DLCT in distinguishing between low- and high-grade BCa.

## Materials and methods

### Patient selection

The institutional review board of our institution approved this retrospective study and waived the requirement for informed consent (Ethical Approval No. I-22PJ887). From October 2017 to October 2019, patients meeting the following criteria were included in the study: (1) having undergone transurethral resection of bladder tumor or radical cystectomy with pathologically confirmed BCa, and (2) availability of preoperative CTU using DLCT within 20 days before surgery. Patients were excluded if they (1) had BCa combined with other tumors; (2) had undergone preoperative therapy, including chemo- or radiotherapy; (3) could not be evaluated due to the lesion being small or invisible; or (4) had poor CTU image quality.

In this study, we performed pathological grading of BCa according to the 2016 World Health Organization (WHO) classification guidelines [3]. The 2016 WHO classification system distinguishes between low- and high-grade BCa based on the morphological appearance of tumor cells under the microscope. Per these guidelines, tumors classified as low-grade display relatively organized cell structures, with mild to moderate variations in nuclear size and shape; those classified as high-grade have markedly abnormal nuclei with significant pleomorphism, hyperchromasia, and an increased number of mitoses. Pathological assessment was conducted by experienced pathologists.

**Fig. 1:**
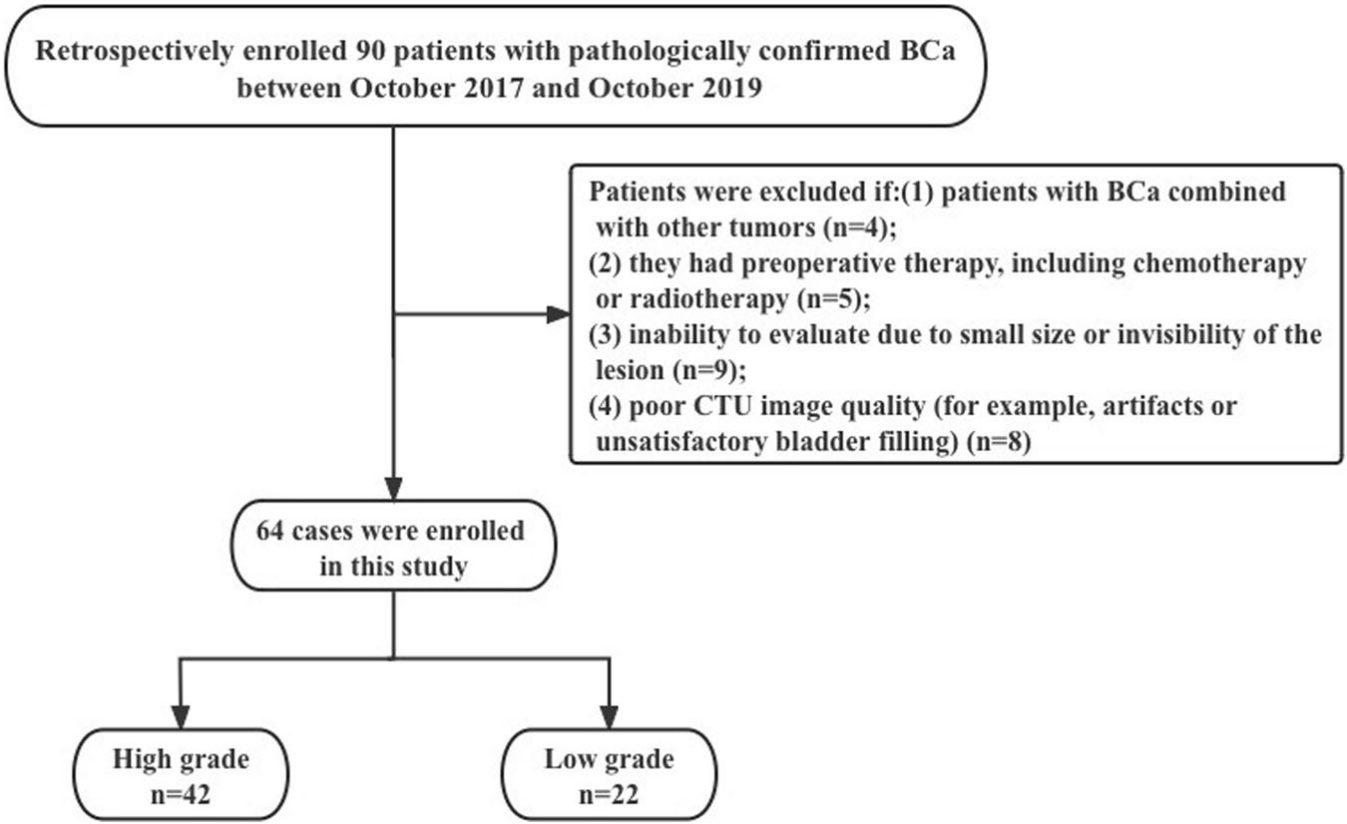
Flowchart of the study

Figure 1 shows the flowchart of patient selection.

### CT protocols

We used a DLCT (IQon Spectral CT; Philips Healthcare, USA) system to perform all examinations, covering the entire abdomen and pelvis. Four-phase images were acquired namely non-enhanced, corticomedullary, nephrographic, and excretory phases. We conducted examinations at a tube potential of 120 kV and detector configuration of 64.0 × 0.625 mm. An intravenous injection of 100 mL of nonionic contrast material (Ultravist 370; Bayer Schering Pharma AG, Germany) was followed by a 100-mL saline chaser at a rate of 4–4.5 mL/s for enhanced scanning. We obtained images of corticomedullary, nephrographic, and excretory phases at 25 s, 75 s, and 300 s, respectively, after injection of the contrast agent. Spectral database image datasets were obtained with a reconstructed slice thickness of 1 mm, spectral reconstruction level set to 3, and an increment of 1 mm. We measured DLCT parameters using the Philips Spectral Diagnostic Suite version 9.0 workstation.

### Image analysis

Two radiologists with 4- and 8-years’ experience in abdominal imaging who were blinded to the pathological results performed all image analyses.

We took quantitative measurements by delineating regions of interest (ROIs). ROIs were delineated along the inside edge of the lesion, and the copy-and-paste function was used to ensure that ROIs were identical in size, shape, and position. When there were multiple lesions, the largest was chosen for data measurements.

**Fig. 2:**
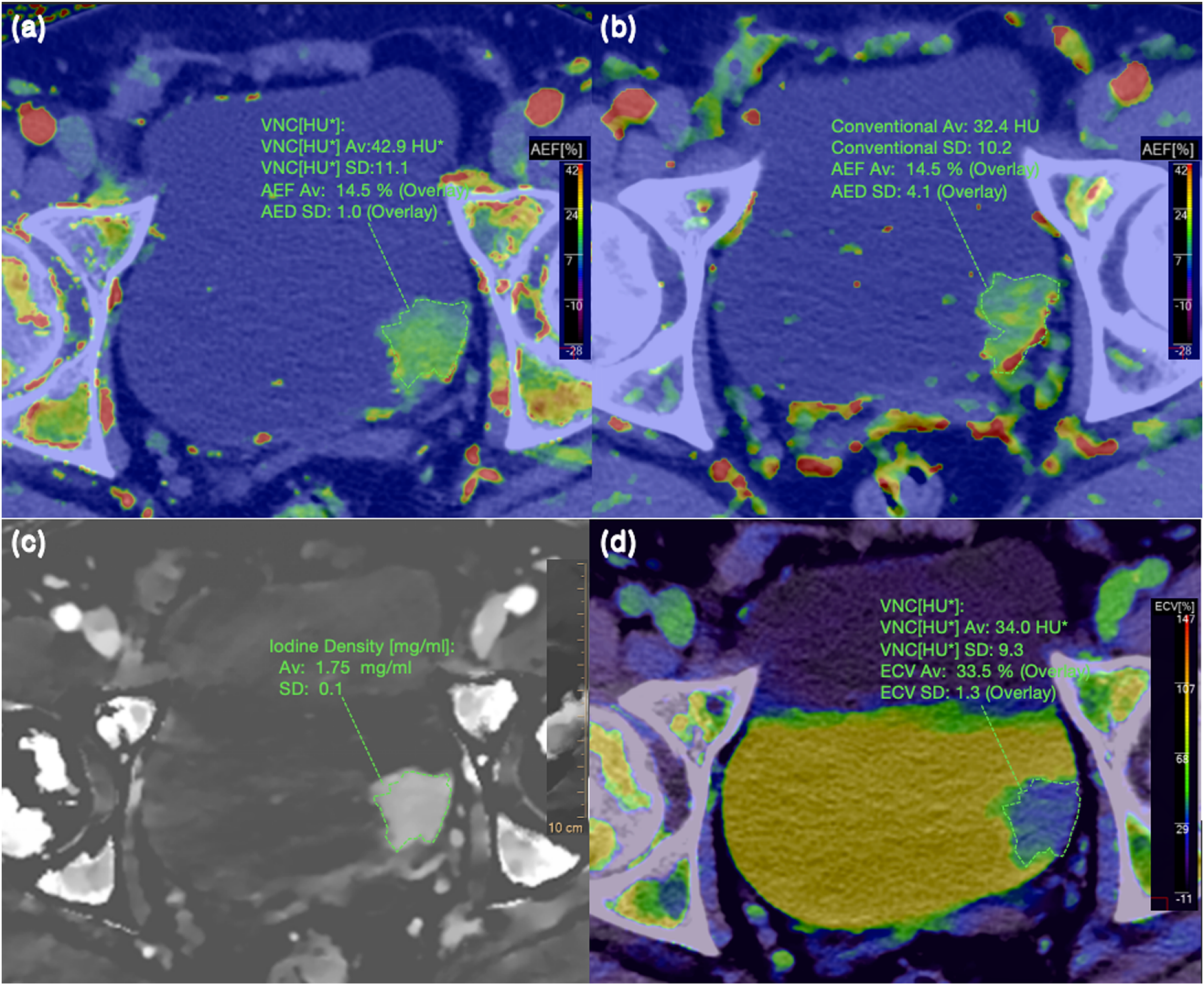
Schematic portrayal of low-grade bladder cancer observed in a 47-year-old man involved in this study. **a** AEF based on ID. **b** AEF based on CT values. **c** ID map. **d** ECV fraction. AEF, arterial enhancement fraction; ID, iodine density; ECV, extracellular volume

Spectral CT parameters were measured as follows (Fig. 2 illustrates some of the measurements):

1. ID (in mg/mL): The dedicated workstation generated IDs of tumors (ID_tumor_) and of the aorta (ID_aorta_). ID_aorta_ was measured at the same level of both renal hila. To minimize the effects of individual circulatory status and scan variations, we divided ID_tumor_ by ID_aorta_ to calculate normalized ID (NID).
  ID-C = ID_tumor_ in corticomedullary phase; ID-N = ID_tumor_ in nephrographic phase; ID-E = ID_tumor_ in excretory phase; NID-C = ID_tumor_/ID_aorta_ in corticomedullary phase; NID-N = ID_tumor_/ID_aorta_ in nephrographic phase; and NID-E = ID_tumor_/ID_aorta_ in excretory phase.
2. Arterial enhancement fraction (AEF) based on ID (AEF-ID) and AEF based on CT values (AEF-HU) were calculated using the following formulas:
  $${{\mathrm{AEF}}}{\mbox{-}}{{\mathrm{ID}}} = \frac{{{\mathrm{ID}}} \, {{\mathrm{cortiomedullary}}} - {{\mathrm{ID}}} \, {{\mathrm{unenhanced}}}}{{{\mathrm{ID}}} \, {{\mathrm{nephrographic}}} - {{\mathrm{ID}}} \, {{\mathrm{unenhanced}}}} \times 100\%.$$
  $${{\mathrm{AEF}}}{\mbox{-}}{{\mathrm{HU}}} = \frac{{{\mathrm{CT}}} \, {{\mathrm{cortiomedullary}}} - {{\mathrm{CT}}} \, {{\mathrm{unenhanced}}}}{{{\mathrm{CT}}} \, {{\mathrm{nephrographic}}} - {{\mathrm{CT}}} \, {{\mathrm{unenhanced}}}} \times 100\%.$$

(3) ECV fraction was calculated using the following formula:
  $$	{{\mathrm{ECV}}} \, {{\mbox{fraction}}}(\%) = (1 - {{\mbox{hematocrit}}})\\ 	 \times \left( \frac{{{\mathrm{ID}}}\, {{\mathrm{in}}}\, {{\mathrm{tumor}}}}{{{\mathrm{ID}}}\, {{\mathrm{in}}}\, {{\mathrm{abdominal}}}\, {{\mathrm{aortic}}}\, {{\mathrm{blood}}}\, {{\mathrm{pool}}}} \right) \times 100\%.$$
  The interval between CT and hematocrit measurement was within the range of 0–7 days.

(4) Slope of the spectral CT-mono-energetic curve (40 and 80 keV; λHU): The slope was defined as the difference of the CT value at 40 and 80 keV divided by the energy difference (80 − 40) according to the following formula:
  $$\lambda \, {{\mathrm{HU}}} = \frac{{{\mathrm{CT}}}(40) - {{\mathrm{CT}}}(80)}{(80 - 40)}.$$

(5) Virtual non-contrast (VNC) images and Z_eff_ images: The dedicated workstation generated VNC and Z_eff_ images based on nephrographic phase.

### Statistical analysis

Spectral CT parameters are presented as means ± standard deviations (SDs) for normally distributed data and as medians (25th–75th percentiles) for non-normally distributed data. We used univariate logistic regression analysis to analyze the significance of spectral CT parameters. Significant parameters were subjected to multivariable logistic regression analysis to obtain the best predictors of BCa histological grade.

We used receiver operating characteristic (ROC) curves, area under the curve (AUC), accuracy (Acc), Sen, Spc, positive predictive value (PPV), negative predictive value (NPV), positive likelihood ratio (+LR), and negative likelihood ratio (−LR) to evaluate the diagnostic performance of parameters identified as significant by multivariable analysis. Cutoff values were used to determine pathological positive outcomes. Decision curve analysis (DCA) was also performed to determine net benefit. Additionally, we calculated the collinearity between AEF-ID and AEF-HU.

Kappa coefficient (*κ*) and intraclass correlation coefficient (ICC) tests were used to test the consistency of the two radiologists’ measurements. We performed statistical analyses using SPSS version 29.0.1.0 (IBM, Armonk, NY), R version 4.3.1 (R Foundation for Statistical Computing, Vienna), and GraphPad Prism version 9.0 (GraphPad Software, San Diego, CA). A two-tailed *p* < 0.05 indicated statistical significance.

## Results

### Clinical data

Ultimately, we excluded four patients from the study due to the presence of other malignancies. An additional five were excluded because they had received neoadjuvant therapy. We excluded nine more patients because their lesions were too small to be identified on CT images. Finally, another eight were excluded due to image artifacts, such as motion artifacts or inadequate bladder filling, which limited visualization. The final study cohort was composed of 64 patients with a mean age of 64 ± 11 years (range, 39–83 years), of whom 42 and 22 were in the high- and low-grade groups, respectively. There were 46 men (64 ± 11 years; range, 39–83 years) and 18 women (64 ± 10 years; range, 41–79 years). Table 1 shows the clinical characteristics of patients.

**Table 1:** Patient clinical characteristics

Clinical characteristic	No. (%)
Gender	
Male	46 (71.9)
Female	18 (28.1)
Age (years)	
< 50	8 (12.5)
50–60	8 (12.5)
60–70	30 (46.9)
> 70	18 (28.1)
Pathological grade	
High	42 (65.6)
Low	22 (34.4)

### Spectral CT parameters

**Fig. 3:**
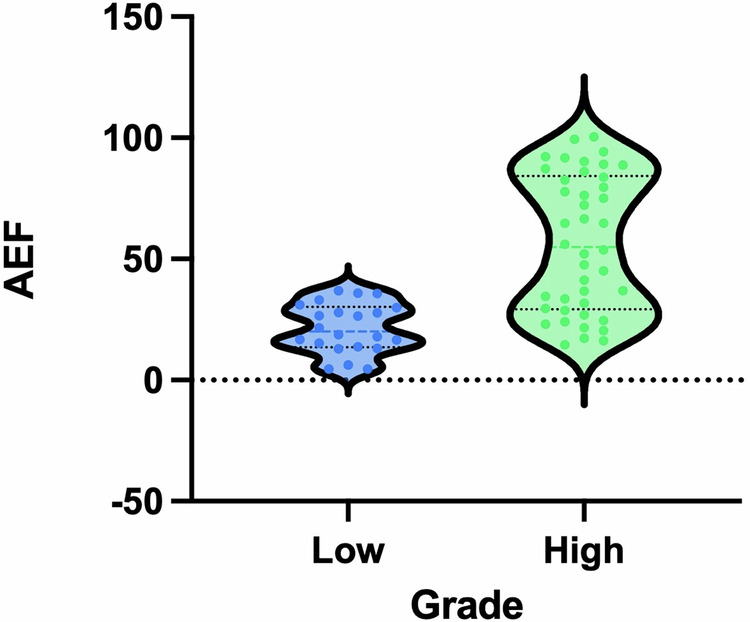
Distribution of AEF-ID in the two groups. AEF-ID, arterial enhancement fraction based on iodine density

**Fig. 4:**
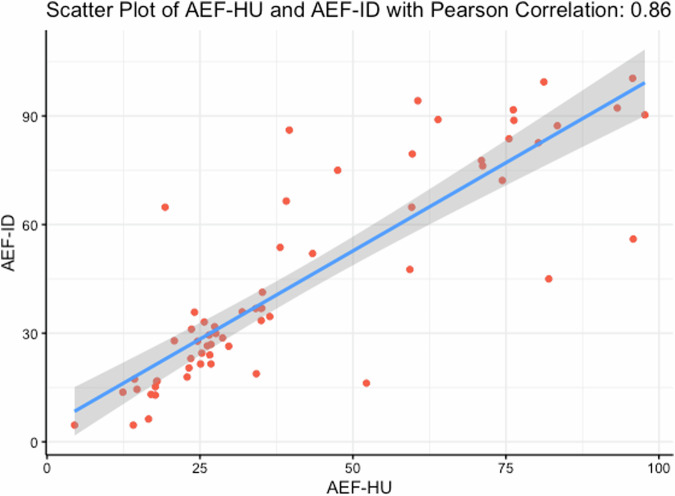
Collinearity results for AEF-ID and AEF-HU. The correlation between AEF-ID and AEF-HU was strong, with a Pearson correlation coefficient (PCC) of 0.86. AEF-ID, arterial enhancement fraction based on iodine density; AEF-HU, arterial enhancement fraction based on CT values

We observed and calculated quantitative spectral CT parameters, which are shown in Table 2. Univariate logistic regression results showed that AEF-ID, ECV, ID-C, NID-C, VNC image, and Z_eff_ image values were significantly associated with tumor grade (*p* = 0.001–0.048; Table 2). Multivariable logistic regression analysis of spectral parameters showed that AEF-ID was significant in both groups (*p* = 0.003; Table 3). AEF-ID demonstrated good predictive performance for BCa grade, with an odds ratio (OR) of 1.089 and a 95% confidence interval (CI) of 1.029–1.153. Figure 3 shows the distribution of AEF-ID for both tumor grades. Collinearity for AEF-ID and AEF-HU was good at 0.86 (Fig. 4).

**Table 2:** Univariate logistic regression analysis of spectral CT parameters

Parameter	Value	Estimate	SD	*Z*	OR (95% CI)	*p*-value
AEF-ID (%)	34.1 (21.5–74.3)	0.096	0.030	3.245	1.101 (1.038–1.167)	0.001*
ECV fraction (%)	28.4 (22.4–33.2)	0.096	0.040	2.415	1.100 (1.018–1.189)	0.016*
ID-C (mg/mL)	1.6 (1.3–2.3)	0.888	0.405	2.191	2.430 (1.098–5.379)	0.029*
ID-N (mg/mL)	2.0 ± 0.7	0.683	0.414	1.649	1.979 (0.879–4.455)	0.099
ID-E (mg/mL)	2.0 ± 0.7	−0.393	0.371	−1.059	0.675 (0.326–1.397)	0.290
NID-C	1.3 (1.0–1.8)	0.141	0.058	2.415	1.151 (1.027–1.290)	0.016*
NID-N	5.4 ± 2.1	0.013	0.014	0.986	1.013 (0.987–1.041)	0.324
NID-E	11.6 ± 4.7	0.010	0.006	1.612	1.010 (0.998–1.023)	0.107
VNC (HU)	35.9 ± 10.6	0.056	0.028	1.981	1.058 (1.001–1.119)	0.048*
*λ*HU (40 keV/80 keV)	3.4 ± 0.7	−0.004	0.041	−0.108	0.996 (0.919–1.078)	0.914
Z_eff_	8.4 ± 0.3	2.403	1.085	2.214	11.052 (1.317–92.764)	0.027*

Values: data are presented as means ± standard deviations (SDs) for normally distributed data and medians (25th–75th percentiles) for non-normally distributed data

ID-C, ID-N, and ID-E: iodine density images in corticomedullary, nephrographic, and excretory phases, respectively; NID-C, NID-N, and NID-E: normalized ID in corticomedullary, nephrographic, and excretory phases, respectively

*AEF-ID* arterial enhancement fraction based on iodine density, *ECV* extracellular volume, *VNC* virtual non-contrast images, *λHU* slope of the spectral curve at 40 and 80 keV, *Z*_*eff*_ Z effective

* *p* < 0.05

**Table 3:** Multivariable logistic regression analysis of spectral CT parameters

	Estimate	SD	*Z*	OR (95% CI)	*p*-value
AEF-ID	0.086	0.029	2.927	1.089 (1.029–1.153)	0.003*
ECV fraction	0.040	0.053	0.754	1.041 (0.938–1.154)	0.451
VNC	0.001	0.054	0.001	1.001 (0.900–1.112)	0.999
ID-C	1.371	0.740	1.852	3.938 (0.923–16.805)	0.064
NID-C	0.024	0.052	0.467	1.025 (0.925–1.136)	0.641
Z_eff_	0.554	1.763	0.314	1.734 (0.055–55.125)	0.753

*AEF-ID* arterial enhancement fraction based on iodine density, *ECV* extracellular volume, *VNC* virtual non-contrast images, *ID-C* iodine density images in corticomedullary phase, *NID-C* normalized ID in corticomedullary phase, *Z*_*eff*_ Z effective

* *p* < 0.05

### Diagnostic performance of significant spectral CT parameters

**Fig. 5:**
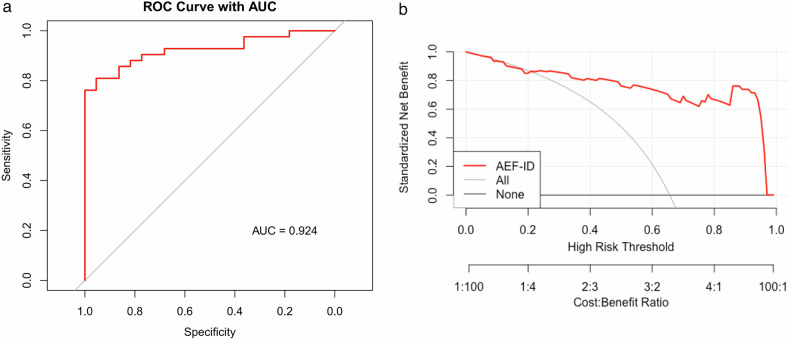
Receiver operating characteristic (ROC) curve (**a**) and decision curve analysis (DCA) (**b**) of AEF-ID in distinguishing high- from low-grade bladder cancer. The ROC AUC was 0.924 (95% CI, 0.861–0.988). The DCA curve shows that using AEF-ID as a predictor of high-grade BCa provided improved net benefits across a range of threshold probabilities. This was particularly true at lower to mid-range thresholds, versus simply categorizing BCa as either high or non-high grade. AEF-ID, arterial enhancement fraction based on iodine density

Table 4 shows the AUC, Sen, Spc, PPV, and NPV of AEF-ID for both groups. AEF-ID had an excellent AUC (0.924) in distinguishing low- from high-grade BCa; its Sen, Spc, Acc, PPV, and NPV were 95.5%, 81.0%, 72.4%, 97.1%, and 85.9%, respectively. Figure 5a shows the ROC curve of AEF-ID in distinguishing low- from high-grade BCa; Fig. 5b shows the clinical decision curve for AEF-ID. As a predictor, AEF-ID demonstrated improved net benefit across a range of threshold probabilities compared with categorizing BCa patients as either high-grade or non-high-grade, particularly at lower to mid-range thresholds.

**Table 4:** Diagnostic efficiency of optimal predictor parameters between groups

Parameter	AEF-ID
ROC AUC (95% CI)	0.924 (0.861–0.988)
Threshold	67.70%
True positive (*n*)	34
False positive (*n*)	1
True negative (*n*)	21
False negative (*n*)	8
Sensitivity (%)	0.955
Specificity (%)	0.810
PPV (%)	0.724
NPV (%)	0.971
Accuracy (%)	0.859
+LR	5.011
−LR	0.056

*ROC AUC* area under the receiver operating characteristic curve, *CI* confidence interval, *AEF* arterial enhancement fraction, *PPV* positive predictive value, *NPV* negative predictive value, *+LR* positive likelihood ratio, *−LR* negative likelihood ratio

### Interreader agreement

Pairwise agreement between the two reader groups in subjective features ranged from κ = 0.707 to κ = 0.913 (*p* < 0.05 for both reviewers).

## Discussion

Our results showed that AEF-ID had excellent diagnostic value in differentiating between high- and low-grade BCa, demonstrating that the use of the spectral parameter AEF-ID to evaluate BCa pathology in diagnosis was effective and instructive. In addition, negative results indicated that other parameters might not be correlated with the pathology of BCa. To the best of our knowledge, this is the first study to evaluate BCa pathology using quantitative parameters obtained with DLCT.

In our multivariable analysis, the OR for AEF-ID was 1.089, with a 95% CI of 1.029–1.153. Although the OR value represented only a slight increase, the narrow CI and the *p*-value of 0.03 indicated that this increase was statistically significant. This suggested that AEF-ID was a meaningful predictor of BCa grade, despite the modest effect size. The narrow CI, which did not include the value 1.000, further underscored the robustness of this association in statistical terms. Given the small sample size in our study, we acknowledge that the observed effect might have been influenced by this limitation. Therefore, the slight increase in the OR value might underestimate AEF-ID’s true predictive power. To address this, we plan to conduct further research with a larger study population to more rigorously assess AEF-ID as a prognostic indicator of BCa grade.

The identified threshold of AEF-ID stood at 67.70%, accompanied by Sen of 95.5%, Spc of 81.0%, and AUC of 0.924. A higher AEF-ID value generally signifies a higher grade of BCa; Fig. 3a illustrates the distribution of the AEF value in both groups. Following are some possible explanations of why AEF performed better than other spectral CT parameters. First, from a hemodynamic perspective, infiltration of cancer cells in BCa induces angiogenesis [26, 27]. This leads to an augmented distribution of contrast agents in the intravascular/extravascular compartment, resulting in an elevated AEF value in the corresponding circulatory phase [28]. Moreover, the use of spectral detector CT in this study enabled concurrent capture of low- and high-energy data at identical spatial and temporal coordinates, resulting in impeccably aligned data and notably diminished measurement error without requiring any pre-determined selection for acquisition mode [29].

We also found good collinearity between AEF-ID and AEF-HU. DLCT, using dual- or multi-energy techniques, can more accurately separate and quantify iodine content in tissues, providing more precise calculations of AEF. However, traditional CT can also be used to obtain AEF values, suggesting that it can be used for analysis in the absence of advanced technologies such as spectral CT. Similar studies, such as that by Huber et al, have found that using AEF based on conventional CT can help screen for hepatocellular carcinoma; Huber et al suggested a cutoff AEF value of 50% [29].

Previous research has suggested that other spectral parameters can serve as valuable tools for assessing different types of cancer. For instance, Fujita et al discovered that ECV fraction could be used to help predict the efficacy of preoperative neoadjuvant chemotherapy in pancreatic ductal adenocarcinoma, which can be attributed to its association with the histological degree of fibrosis and quantity of desmoplastic stroma [30]. We hypothesized that ECV fraction could help differentiate between high- and low-grade BCa. In our univariate analysis, ECV fraction showed significance, while in multivariable analysis, it was not a significant parameter. This indicated that ECV fraction might lack the requisite statistical strength to be considered a robust differentiating factor for BCa grade in a more comprehensive prognostic model.

VNC images can provide baseline CT values just like true non-contrast images, which can differentiate tumors from surrounding normal tissues. The distinct appearances of tumors in pre- and post-contrast images can be more clearly revealed by VNC imaging, aiding in tumor identification and staging. Zhang et al showed that VNC images can maintain good image quality, permitting a decrease in radiation dose in the diagnosis of renal cell carcinoma [31]. Wang et al found that Z_eff_ has high Sen and Spc for predicting pathological subtype and risk stratification of ground-glass nodules (GGNs), perhaps because the pathological subtypes of different GGNs are composed of different substances and can be reflected by Z_eff_ [32]. Tanaka et al found that low-ID tumor area ratio was a useful prognostic index of non–small-cell lung cancer after stereotactic body radiotherapy [33], possibly due to tumor tissues typically having abnormal hemodynamic perfusion characteristics. Malignant tumors are often neovascularized, leading to increased blood flow. ID can help identify these differences in perfusion and assess the blood supply to the tumor.

Additionally, λHU reflects how the attenuation of various substances varies with the energy distribution of X-ray photons. This variation is linked to the composition and density of the substances, as well as to interactions between the photon energies and the substances themselves [25]. Unfortunately, our study did not identify the λHU as a strong predictor of BCa pathology. Further analysis of BCa’s cellular composition and pathological aspects might be necessary to clarify the underlying reasons. Alternatively, the negative results we observed could be ascribed to the limited sample size.

Our study had some limitations. First, due to circumstances beyond our control, our access to the Philips IQon CT scanner was limited to the period from October 2017 to October 2020 for routine CT scanning in our department; hence, this was a single-center exploratory study with a small sample size. Second, the spectral CT features we examined were not comprehensive, nor were they combined with conventional CT features. Certain crucial features might have been omitted, thereby necessitating future investigations to encompass a broader range of CT features for analysis. Third, not all pathological sections might have been matched with imaging ROIs, potentially introducing bias into the analysis.

In summary, the spectral parameter AEF-ID proved to be a significant prognostic factor in BCa grading, despite its modest effect size. We plan to validate its predictive value in a larger study population in subsequent studies. This approach could help clinicians devise initial treatment strategies, potentially improving patient outcomes.

## Data Availability

The data are available upon request.

## Abbreviations

AEF: Arterial enhancement fraction
BCa: Bladder cancer
DECT: Dual-energy computed tomography
DLCT: Dual-layer spectral detector computed tomography
ECV: Extracellular volume
ID: Iodine density
NID: Normalized iodine density
VNC: Virtual non-contrast
Z_eff_: Z effective

## Materials availability

The materials are available upon request.
